# Seagrass Colonization Alters Diversity, Abundance, Taxonomic, and Functional Community Structure of Benthic Microbial Eukaryotes

**DOI:** 10.3389/fmicb.2022.901741

**Published:** 2022-06-13

**Authors:** Ying Pan, Guihao Li, Lei Su, Pengfei Zheng, Yaping Wang, Zhuo Shen, Zigui Chen, Qiuying Han, Jun Gong

**Affiliations:** ^1^School of Ecology, Sun Yat-sen University, Shenzhen, China; ^2^Laboratory of Microbial Ecology and Matter Cycle, School of Marine Sciences, Sun Yat-sen University, Zhuhai, China; ^3^Yantai Institute of Coastal Zone Research, Chinese Academy of Sciences, Yantai, China; ^4^Department of Microbiology, The Chinese University of Hong Kong, Hong Kong, China; ^5^College of Ecology and Environment, Hainan Tropical Ocean University, Sanya, China; ^6^Southern Marine Science and Engineering Guangdong Laboratory, Zhuhai, China; ^7^Guangdong Provincial Key Laboratory of Marine Resources and Coastal Engineering, Guangzhou, China

**Keywords:** community structure, belowground diversity, protist, parasite, high throughput sequencing, functional composition

## Abstract

Seagrass form high productive ecosystems in coastal environments. However, the effects of these coastal plants on the structure and function of the belowground eukaryotic microbiome remain elusive. In this study, we characterized the community of microbial eukaryotes (microeukaryotes) in both vegetated and unvegetated sediments using 18S rRNA gene amplicon sequencing and quantitative PCR. Analysis of sequencing data showed that the eelgrass (*Zostera marina*) colonization decreased the alpha diversity indices of benthic microeukaryotes. Apicomplexa represented an average of 83% of reads across all samples, with a higher proportion at the vegetated sites. The taxonomic community structure was significantly different between these two types of sediments, for which the concentration of ${\text{NH}}_{4}^{+}$ in sediment porewater and salinity could account. Phylogenetic analyses of long 18S rRNA genes (around 1,030 bp) indicated these apicomplexan parasites are closely related to gregarine *Lecudina polymorpha*. Determination of 18S rRNA gene abundances provided evidence that the eelgrass markedly promoted the biomass of the gregarine and all microeukaryotes in the seagrass-colonized sediments and confirmed that the gregarine was hosted by a polychaete species. Significantly higher gene abundances of heterotrophs and mixotrophs were found at the vegetated sites, which could be explained by the finer sediments and short supply of dissolved inorganic nitrogen, respectively. The pigmented protists were more abundant in 18S rRNA gene copies at the lower and higher pH levels than at the intermediate. Nevertheless, the fractions of heterotrophs and phototrophs in the community were significantly related to porewater N:P ratio. These results indicate that seagrass colonization significantly induces an increase in overall biomass and a decrease in diversity of benthic microeukaryotes, making them more heterotrophic. This study also highlights that the hotspot of eukaryotic parasites could be linked with the high productivity of a natural ecosystem.

## Introduction

Seagrass meadows in shallow estuarine and coastal environments are globally important ecosystems that provide habitats for thousands of fish, bird, and invertebrate species (Han et al., 2017; McKenzie et al., 2020). Seagrass trap particulates from the overlying water, deposit and bury tissue detritus, release dissolved organic matter and dissolved oxygen through roots, which fuel macro- and microorganisms, and modify geochemical characteristics of the sediments they colonize (Garcias-Bonet et al., 2012). Seagrass colonization has been demonstrated to not only enhance the abundance of microbes such as bacteria, archaea, and some specific prokaryotic lineages, but also their diversity in the sediments (Sun et al., 2015; Zheng et al., 2019; Lin et al., 2021). The microbial communities in vegetated sediments could in turn support seagrass productivity and maintain health *via* mediating carbon, nitrogen, and sulfur cycling, and oxidizing potentially toxic sulfides (Ikenaga et al., 2010; Sun et al., 2015; Wang et al., 2021). Nevertheless, recent advances in studying seagrass-associated microbial diversity are mainly restricted to prokaryotes, and little is known about the microbial eukaryotes (microeukaryotes).

The effects of seagrass on quantity and community structure of microeukaryotes in the sediments they colonize are perceivable, but remain to be experimentally investigated. From a functional point of view, seagrass colonization may lead to a more heterotrophic microeukaryotic community because seagrass leaves shelter surface sediment from light, which could limit the growth and biomass of microalgae (microphytobenthos) on the sediment surface (MacIntyre et al., 1996). Furthermore, the higher prokaryotic abundance in vegetated sediments supplies more food for bacterivorous protists (Zheng et al., 2019), thus promoting the proportions and biomass of heterotrophs, such as flagellates, ciliates, and amoebae (Fenchel, 1969). Using molecular approaches, recent surveys on diversity of marine microeukaryotes have detected abundant protistan parasites in productive systems, such as deep-sea hydrothermal vents (Moreira and López-García, 2003), neritic oceans (Gong et al., 2015), and polar marine environments (Liu et al., 2021). Given the cryptic nature of these microparasites and the general importance of parasites in trophic interactions affecting food web structure, keystone species, and ecosystem processes (Preston and Johnson, 2010; Fischhoff et al., 2020), the identities of microeukaryotic parasites and their hosts in sedimentary systems remain to be revealed and hypotheses regarding distributional patterns and regulating factors (such as the relationship with local primary productivity) have yet to be tested.

In this study, we aimed to characterize the diversity, taxonomic, and functional composition of benthic microeukaryotes in a seagrass system using sequencing of 18S rRNA genes. By comparing the vegetated and unvegetated sediments, we tested the hypothesis that seagrass colonization enhanced the diversity and quantity of benthic microbial eukaryotes and altered their taxonomic and functional composition. We detected a predominant apicomplexan parasite in these communities, which raised the question of its phylogenetic position, quantity, and distribution across sediment samples. We also assessed whether it was associated with migrating swans or infauna species. Our results support that the microeukaryotic community tends to be more heterotrophic in the seagrass-colonized sediments and that the apicomplexan parasite may be hosted by a polychaete species which tends to be more abundant in seagrass-colonized sediments.

## Materials and Methods

### Study Area, Sampling, and Environmental Parameters

The seagrass system was located at the Swan Lake (Tian'ehu) lagoon (37°21′N, 122°35′E), Rongcheng Bay, Yellow Sea, northern China. This lagoon is a typical temperate habitat with eelgrass (*Zostera marina*) meadows developed properly during both the spring and summer seasons and is known as the largest swan overwintering habitat in Asia (Sun et al., 2015). Owing to the vegetation of seagrass, the lagoon is rich in biological resources such as shellfish, fish, and other benthic macro-organisms, which serve as food sources for ~10,000 of whooper swans (*Cygnus cygnus*) that overwinter every year (Sun et al., 2020).

The sampling strategy and characterization of physiochemical parameters of water and sediments were described previously as in Sun et al. (2015). Briefly, on a day of May 2013, we sampled five sites (referred to as V1–V5 thereafter) from within an area covered by the eelgrass (*Zostera marina*) meadow, and another five sites (UV1–UV5) ~20–40 m distant from the vegetated area but not vegetated. The top layer (~5 cm) of the sediments was collected and stored at −80°C for DNA extraction. The temperature, pH, salinity, and concentrations of dissolved oxygen (DO) and chlorophyll *a* (Chl-*a*) of the overlying water, and the median diameter of the sediment particles, the concentrations of ammonium (${\text{NH}}_{4}^{+}$), nitrate (${\text{NO}}_{3}^{-}$), nitrite (${\text{NO}}_{2}^{-}$), and soluble reactive phosphate (${\text{PO}}_{4}^{3-}$) in the sediment porewater, the total organic carbon (TOC) and total organic nitrogen contents (TON), and the concentrations of metals at each site were characterized and reported in Sun et al. (2015).

After phylogenetically classifying the dominant apicomplexan parasite (*Lecudina*, see below), we were interested in the source hosts of this parasite. Instead of examining a wide range of animal candidates, we focused on two suspect species, the migrating swans, which regularly visit the lagoon every year with high abundance, and a polychaete species (*Lumbrineris latreilli*), since there are records for *Lecudina* isolates from sandworms (Leander et al., 2003). Fresh and undisturbed swan feces were collected at high tidal sites with a clean spade, put on ice, transported to the laboratory, and stored at −80°C. The live specimens of *Lumbrineris latreilli* were isolated from the sediments colonized by the eelgrass. The worms were washed with seawater and then sterilized seawater on site for several times to minimize contamination, put into liquid nitrogen, and then stored at −80°C until DNA extraction.

### DNA Extraction and Microeukaryotic 18S rRNA Gene Amplicon Sequencing

The extractions of DNA from 0.5 to 0.7 grams of sediments, swan feces, and *Lumbrineris latreilli* specimens were performed using the FastDNA spin kit for soil (MP Biomedical, USA) according to the manufacturer's instruction. The DNA integrity was verified on a 1.5% agarose gel, and the DNA purity and concentration were determined using an ND-2000C spectrophotometer (NanoDrop, USA). The extracted DNA was stored at −80°C for later use.

For high-throughput sequencing, the DNA of both vegetated (V1–V5) and unvegetated (UV1–UV5) sediment samples was sent to Novogene (Beijing, China) for MiSeq sequencing using the eukaryote-universal primers 82F (5′-GAAACTGCGAATGGCTC-3′, López-García et al., 2001) and 516R (5′-ACCAGACTTGCCCTCC-3′, Amann et al., 1990). Each sample was identified with a specific 6-bp barcode in the reverse primers. Each 30 μL of PCR reaction solution contained 2 × Phusion High-Fidelity PCR Master Mix (New England Biolabs), 0.2 μM of each primer, and 10 ng of DNA. The PCR was performed with a T100 Thermal Cycler (Bio-Rad, USA) with an initial denaturation at 98°C for 1 min, followed by 30 cycles of denaturation at 98°C for 10 s, annealing at 50°C for 30 s, and elongation at 72°C for 30 s, with the last extension step at 72°C for 5 min. Paired-end sequencing was performed with an NEB Next Ultra DNA Library Prep Kit for Illumina on the Illumina MiSeq platform (Illumina, USA) by an outside company (Novogene, Beijing, China).

### Bioinformatic Analysis of Microeukaryotic 18S rRNA Gene Short Reads

Raw data of MiSeq sequencing were processed and analyzed based on QIIME v.1.9.0 (Caporaso et al., 2010) and Mothur v.1.35.1 (Schloss et al., 2009). Quality filtering was according to the following criteria: quality score > 20; no ambiguous bases; no primer sequence mismatches; and homopolymers ≤ 6. Chimera checking was run with USEARCH v.61 based on the SILVA database (119 release), and the putative chimeric sequences were removed. After quality filtering of 353,164 raw reads of 18S rRNA genes, a total of 203,339 reads retained were clustered into operational taxonomic units (OTUs) at 97% sequence similarity with UCLUST v.1.2.2 (Edgar, 2010), and representative sequences of the OTUs were extracted to assign taxonomic classification against the PR^2^ database (Guillou et al., 2013). Singletons (the OTUs containing a single read across all samples) were excluded prior to further analysis. The reads of macro-organisms were discarded before subsequent analyses (Zhu et al., 2018).

To normalize the sampling effort, datasets were rarefied to the minimal number of reads across the samples (8,710 sequences) for microeukaryotes. We calculated alpha diversity estimators including OTU richness, Chao1, Simpson, and Shannon indices. Bray–Curtis dissimilarity index-based beta diversity was calculated and visualized using non-metric multiple dimensional scaling (nMDS) in the package PRIMER v.6.0 (Clarke and Gorley, 2006). The ANOSIM function within PRIMER was performed to test the hypothesis that microeukaryotic community was different between the vegetated and unvegetated sediment sites. The contributions of taxonomy groups to the community dissimilarity between these two sites were calculated using the function SIMPER. Redundancy analysis (RDA) was used to explore correlations between environmental factors and variations in communities with CANOCO v.4.5 with a Monte Carlo permutation test (999 permutations) (ter Braak and Smilauer, 2002).

### Microeukaryotic 18S rRNA Gene Cloning and Sanger Sequencing

Longer 18S rRNA gene fragments (~1,030 bp) of microeukaryotes, which provide generally finer resolution of taxonomic classification of eukaryotic species, were obtained for sediment and feces samples *via* clone libraries and Sanger's sequencing. The eukaryote-universal primers E528F: 5′-CGGTAATTCCAGCTCC-3′; Edgcomb et al., 2002) and 1391RE (5′-GGGCGGTGTGTACAARGRG-3′; Dawson and Pace, 2002) were used in PCR reactions. Each reaction solution (25 μl) contained 2.5 μl 10 × *Taq* buffer, 0.5 μl dNTP (2.5 μM), 1 μl of each primer (10 μM), 0.125 μl *Taq* polymerase (5 U μl^−1^), 1 μl DNA template, and 18.875 μl ddH_2_O (Thermo Scientific, USA). The PCR amplification program was as follows: an initial denaturation at 94°C for 5 min, 30 cycles of denaturation at 94°C for 60 s, annealing at 56°C for 60 s, and elongation at 72°C for 150 s, with a final elongation at 72°C for 10 min. PCR products derived from the five sediment samples were pooled to generate a clone library for the vegetated (V_Clone) and the unvegetated (UV_Clone), respectively. Another library was prepared for the fecal samples of swans. The PCR products were purified using a TIANprep Midi Purification Kit (Tiangen, Beijing, China), and clone libraries were constructed using InsTAclone PCR Cloning Kit (Thermo Scientific, USA). The positive clones were subjected to colony PCR with primers M13F/M13R; the PCR products were further analyzed using restriction fragment length polymorphism (RFLP) with restriction enzymes *Msp*I and *Hal*I (Thermo Scientific, USA). Digestion was conducted on 37°C water bath for 2 h, and the products were checked on 2% agarose gels to identify RFLP types. Several clones of each RFLP type were selected for Sanger sequencing, which was performed in both directions on ABI 3700 sequencer (Sangon, Shanghai). We randomly sequenced 96 and 124 positive clones for vegetated and unvegetated sediment samples, respectively.

### Phylogenetic Analysis of 18S rRNA Gene Sequences

Environmental sequences from the sediment and swan feces clone libraries were checked to remove possible chimers using Bellerophon (Huber et al., 2004). For sediment clone library, OTUs were determined at 97% cutoff with the Mothur package. After removing putative chimeric sequences, 77 and 86 sequences were retained for vegetated and unvegetated sediment samples, respectively. Representative sequences of microeukaryote and possible gregarine OTUs identified by BLASTn appended with closely related sequences derived from GenBank were used in phylogenetic analyses. The sequences were aligned using MAFFT (Katoh and Standley, 2013), and the alignment was manually adjusted. A maximum likelihood (ML) tree was constructed by RaxML v8.2, with an appropriate model GTRGAMMAI and 1,000 bootstrap analysis (Stamatakis, 2014). A Bayesian inference (BI) tree was constructed using MrBayes v. 3.2.6 with a GTR + I +G model (Ronquist et al., 2012). Four MCMC chains were run for 3,000,000 generations and sampled every 100 generations. The first 750,000 generations were discarded as burn-in. For the swan feces clone library, sequences from each RFLP type were identified by BLASTn to get closely related microeukaryote species that were morphologically identified. A pie was drawn according to the proportion of each type by Microsoft Excel to recover the microeukaryote community structure in swan feces.

### Quantitative Real-Time PCR (qPCR) of *Lecudina polymorpha*

Specific primers of the species *Lecudina polymorpha* (Apicomplexa, Gregarines), for which the 18S rRNA gene fragments were found to be very abundant in the eukaryotic microbiome in this study, were newly designed and verified as previously described (Su et al., 2018). Briefly, the primers were first evaluated *in silico* using probeCheck and further tested by constructing clone library for seagrass sediment environmental DNA. The PCR program for the primer set 651F/Gre736R was as follows: 94°C for 5 min, 30 cycles of 94°C for 1 min, 57°C (55°C for Gre1138F/Gre1378R) for 1 min, 72°C for 30s, and a final extension at 72°C for 10 min. Clone library screening and Sanger sequencing were as above described. Bellerophon was used to identify chimeras in newly obtained environmental sequences. A maximum likelihood (ML) tree consisting 13 gregarine sequences retrieved from GenBank and the 69 sediment clone sequences used in primer design was built using FastTree (Price et al., 2009) to check the phylogenetic positions of the resulting sequences derived from qPCR assays.

The qPCR assays were performed with SYBR Green Dye on an ABI 7500 Fast Real-Time PCR System (Applied Biosystems, USA) with the new primer set Gre651F/Gre736R to obtain the *L. polymorpha* rRNA gene copy numbers in seagrass sediment and sandworm. All the reactions were performed in triplicate. The *L. polymorpha* rRNA gene copy numbers were calculated using standard curves generated *via* multiple serial dilutions (10^−1^-10^−10^) of the plasmid standards with inserted target rRNA gene fragments.

### Functional Assignments

The functional structure of microbial eukaryotes was examined using a trait-based approach (Wang et al., 2020). In brief, the functional assignments of community members were transformed from taxonomic assignments by PR2 based on annotations of ecological (functional) traits of relatively higher ranked eukaryotic groups (Adl et al., 2019), with retaining their “abundance” information (i.e., proportions of reads) in a given community. The read proportion of the taxa assigned into a specific functional category in a community was summed to represent the “relative abundance” of that functional group, which allowed to quantitatively examine how eukaryotic functions varied across samples. In this study, three functional categories were assigned: autotrophs, mixotrophs, and heterotrophs, which were further divided into parasites and phagotrophs. To understand how the functional structure of microbial eukaryotes was organized, read proportions and calculated 18S rRNA gene abundances of these functional groups were compared between the two types of sediments and along environmental gradients.

### Statistical Analysis

*T*-test was performed to statistically test the difference in relative and absolute abundances of major taxonomic or functional groups between vegetated and unvegetated samples. Spearman's and Pearson's correlation coefficients were calculated to explore the associations between richness, read proportions and 18S rRNA gene copy numbers of entire microeukaryotic community, individual taxa (including the dominant parasite *Lecudina polymorpha*), functional groups, and environmental factors using the package SPSS v.11.5.

## Results

### Taxonomic Diversity and Community Composition of Benthic Microbial Eukaryotes

**Figure 1:**
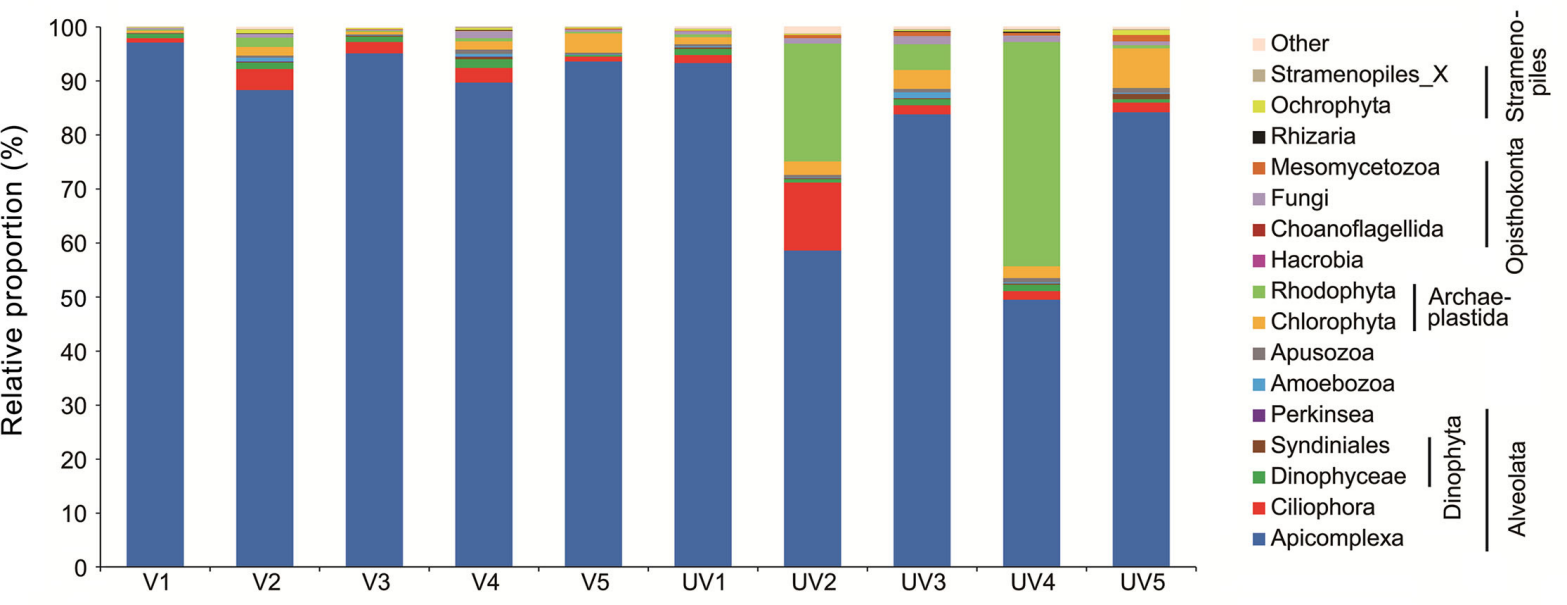
Relative proportions of reads for benthic microbial eukaryotes across vegetated (V1–V5) and unvegetated (UV1–UV5) sites of the seagrass ecosystem.

For all 10 samples collected from both sites, macro-organisms such as Metazoa (mainly Nematoda; on average 11.7%) and Streptophyta (mainly the eelgrass *Zostera*; on average 16.5%) accounted for highly variable proportions ranging from 0.1 to 65.4% and from 1.5 to 22.4%, respectively (Supplementary Figure S1). To inspect microbial eukaryotes, these reads from metazoan and eelgrass debris were discarded (Figure 1), resulting in a total of 472 OTUs for single-celled eukaryotes. Reads of Apicomplexa dominated the communities, accounting for 83.2% of the total sequences, with the proportions ranging from 9.4 to 97% (Figure 1). Read proportions of Rhodophyta (0.2–41.5%; mean 7.2%), Ciliophora (0.8–12.6%; mean 3%), Chlorophyta (0.4–7.3%; mean 2.4%), and Dinophyta (0.4–1.9%; mean 1.2%) were followed. Apart from Apicomplexa, a clear dominance of phylum Rhodophyta was observed in samples UV2 and UV4, which reflected large variability of microeukaryotic community among the unvegetated sites. Other groups such as Fungi (0.8%), Mesomycetozoa (0.4%), and Ochrophyta (0.4%) were minor (Figure 1).

**Figure 2:**
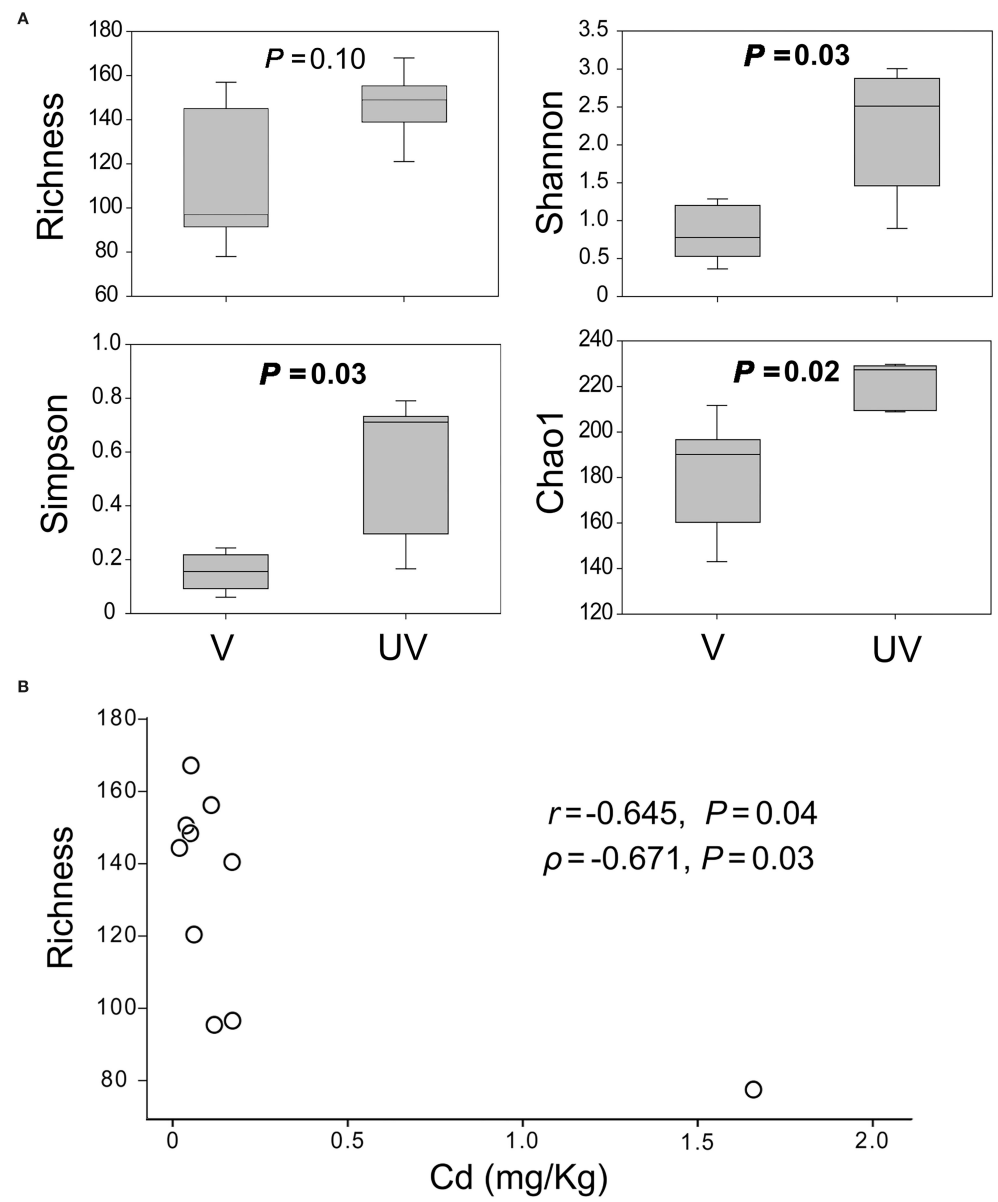
**(A)** Alpha diversity indices of benthic microeukaryotic OTUs, showing the Shannon, Simpson, and Chao1 estimators of microeukaryotes were significantly higher in the unvegetated than in the seagrass-colonized sites at *P* < 0.05 according to *t*-test; **(B)** a negative correlation between microeukaryotic OTU richness and concentration of Cd.

The alpha diversity estimators of microeukaryotic OTUs (Shannon, Simpson, and Chao1) were significantly higher in the unvegetated sediments than the vegetated ones (*P* < 0.05), except for OTU richness (*P* = 0.10; Figure 2A). Among all measured environmental factors, the concentration of metal Cd was most significantly and negatively correlated with OTU richness of microeukaryotes (Pearson's *r* = −0.645, Spearman's ρ = −0.671, *P* < 0.05; Figure 2B). Negative correlation between the concentration of Arsenic (As) and OTU richness was significant as well (*r* = −0.624, *P* = 0.05, but ρ = −0.491, *P* = 0.15) across all these 10 samples (Supplementary Figure S2).

**Figure 3:**
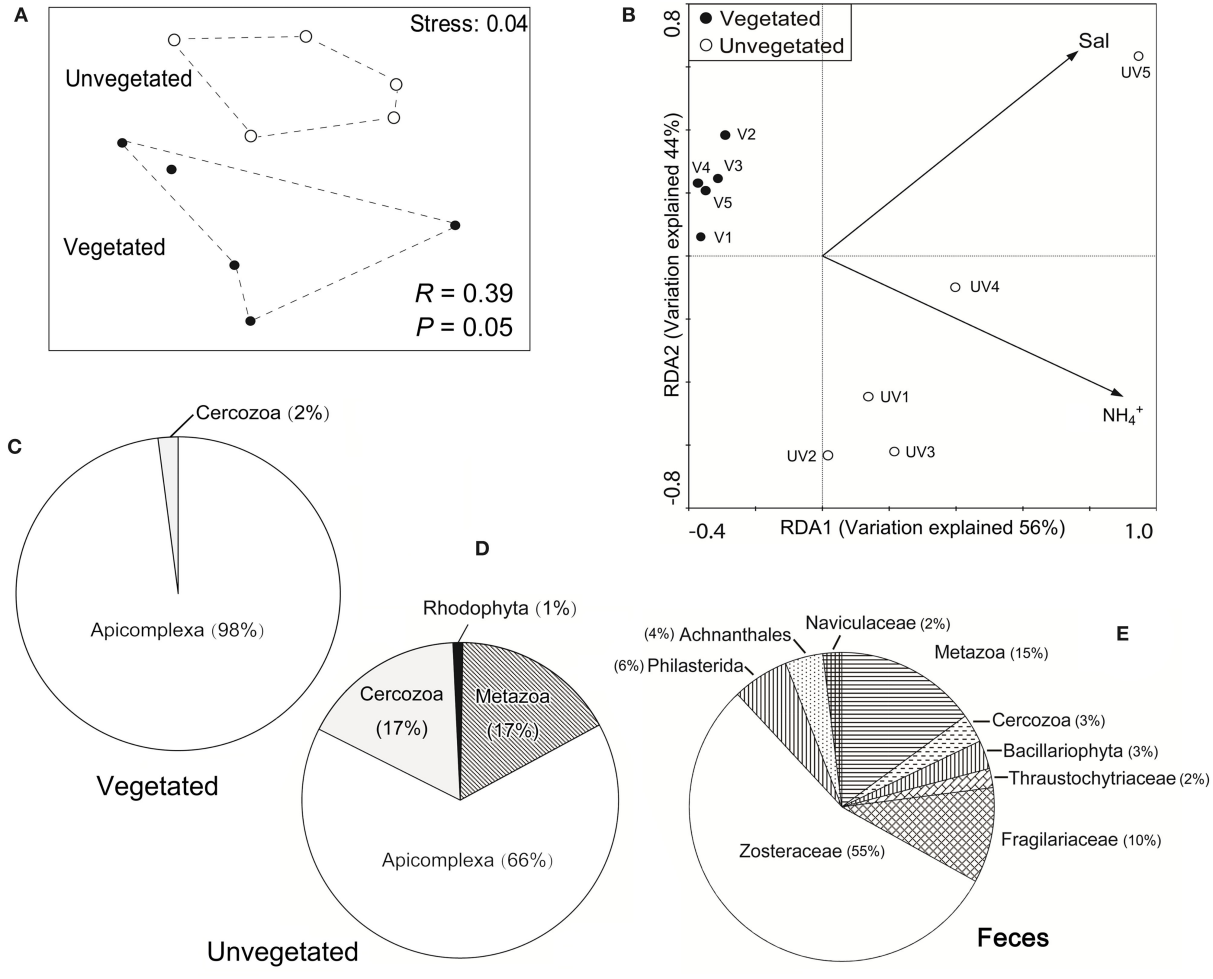
**(A)** A plot of non-metric multidimensional scaling, showing that the community structure of benthic microeukaryotes was significantly different in the vegetated and unvegetated sediment types; **(B)** a plot of redundancy analysis (RDA), indicating that the changes in microeukaryotic community structure covaried significantly with the concentration of ammonium and salinity across all the 10 sites (Monte Carlo tests, *P* < 0.05); and **(C–E)** pie plots showing the sequence proportions of major eukaryotic taxa based on 18S rRNA gene clone libraries derived from the vegetated **(C)**, unvegetated **(D)**, sediments, and from swan feces **(E)**.

The nMDS plot showed that the microeukaryotic communities in the vegetated sediments were markedly different from those in the unvegetated (Figure 3A), which was statistically supported by ANOSIM (R = 0.39, *P* = 0.048). The plot of RDA showed that the concentration of ${\text{NH}}_{4}^{+}$ was the most important environmental parameter affecting the microeukaryotic community across seagrass and bare sediments (*P* < 0.05), and salinity was the factor explaining the community heterogeneity within the unvegetated sediment group (*P* < 0.05) (Figure 3B).

### Differences in Community Structure Between Sediment Types and Contributing Lineages

SIMPER analysis could quantify the contribution of each group of microeukaryote to the community difference (Supplementary Table S1). The contribution of fungi to the microeukaryotic community difference between two sediment types reached 16.48%, which was the highest among all taxonomic groups of microeukaryotes, though the average read proportion of all fungi was only about 0.8%. This is likely due to distinct patterns of OTU presence between these two types of sediments as reflected by the high richness of fungi (87, Supplementary Table S1). Their presence or absence matters for the community, in particular if some fungal OTUs consistently existed at the vegetated sites, whereas other OTUs were absent, and vice versa. The Chlorophyta, Dinophyta, and Ciliophora, whose read proportions were on average 2.4, 1.2, and 3.0%, were responsible for 14.67, 12.68, and 12.25% of microeukaryotic community differences, respectively. Although the read proportion of Apicomplexa was up to 83.2% in the microeukaryotic communities, their contribution was only about 9.58%.

The read proportions of most eukaryotic lineages showed no substantial differences between these two types of sediments (*P* > 0.05), except for a few subgroups (Figure 1B; Supplementary Table S2). These included gregarines and tintinnid ciliates, which represented 91.96 and 0.18% reads in the vegetated samples and were significantly higher than their proportions (73.78 and 0.02%) in the unvegetated sediments (*P* ≤ 0.05). In contrast, the reads of Trebouxiophyceae (0.26 vs. 0.54%), Opisthokonta (0.66 vs. 1.64%), and Mesomycetozoa (0.08 vs. 0.64%) appeared to be more abundant in the unvegetated sediments (*P* < 0.05).

### Eukaryotic Diversity Based on Clone Libraries of 18S rRNA Genes

Both ML and BI trees showed that most (82%) of these non-*Zostera marina* 18S rRNA genes were affiliated to phylum Apicomplexa, whose proportion was higher in the vegetated (98%) than in the unvegetated samples (66%) (Figures 3C,D; Supplementary Figure S3), which was consistent with the MiSeq dataset (Figure 1B). Similarly, other 18S rRNA gene sequences belonging to cercozoans (mainly Plasmodiophoridae and Thaumatomastigidae; 17%) and benthic animals [namely genera *Enoplus* and *Oncholaimus*, family Enchelidiidae (Nematoda), family Polycystididae (Platyhelminthes), and family Loxoconchidae (Arthropoda)] were also highly represented in the unvegetated sediments (17 vs. <2% in the vegetated; Figures 3C,D; Supplementary Figure S3).

**Figure 4:**
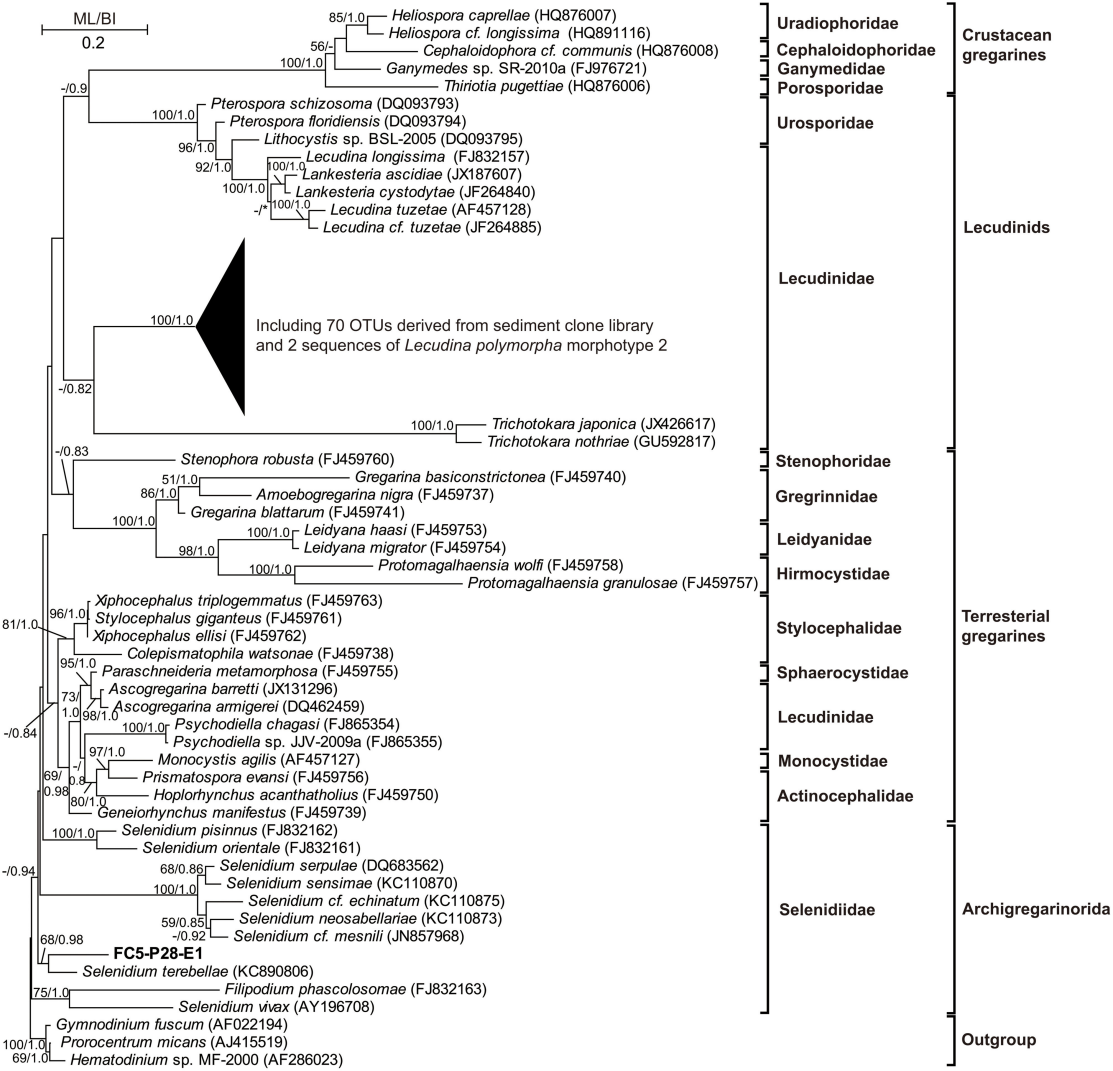
A phylogenetic tree based on 18S rRNA genes and constructed using maximum likelihood (ML) and Bayesian inference (BI) algorithms, showing that most of the gregarine (Apicomplexa) sequences/OTUs obtained from the clone libraries are closely related to that of a described species, *Lecudina polymorpha* morphotype 2 (family Lecudinidae). A few sequences representing a single OTU are affiliated with the genus *Selenidium* (family Selenidiidae). Only bootstrap values >50 and posterior probability >0.8 are shown at the nodes; the lower values are indicated with dashes. Asterisks refer to alternate topologies.

The 18S rRNA genes in swan feces examined using clone library analysis showed a markedly different eukaryotic community from those in sediments (Figure 3E). The gut content of swans included the eelgrass (55% in sequence proportion in the library), Metazoa (15%), Fragilariaceae (10%), Philasterida (6%), Achnanthales (4%), Bacillariophyta (3%), Cercozoa (3%), Naviculaceae (2%), and Thraustochytriaceae (2%), but no Apicomplexa (Figure 3E). Most of the apicomplexan sequences (81%) had high similarities (92–94%) with that of a single described gregarine species, *Lecudina polymorpha* morphotype 2, which belonged to the family Lecudinidae, order Eugregarinorida. Sequences of another gregarine family Selenidiidae (Eugregarinorida) were also detected, but rare in the clone libraries (1%; see Figure 4; Supplementary Figure S3).

### Quantification of *Lecudina polymorpha* Using QPCR

To inspect the “real abundance” of *Lecudina polymorpha*, two species-specific primer pairs, Gre651F–Gre736R and Gre1138F–Gre1378R, were newly designed for quantifying its 18S rRNA genes in environmental samples (Table 1; Supplementary Figure S4). The specificity of these primers was validated by *in silico* evaluating, cloning, sequencing, and classifying the amplicons using the environmental DNA extracted in this study (Supplementary Figure S5).

**Table 1:** Primers newly designed for targeting 18S regions of rRNA genes in gregarines.

**Primer name**	**Sequence (5^**′**^-3^**′**^)**	**Length**	**GC%**	**Tm (**°**C)**	**References**
Gre651F	GCATGGAAYACAGAATTGG	19	44.7	48.7–51.9	This study
Gre1138F	TAGGTCATAGTAACTTGGAG	20	40	47.9	This study
Gre736R	GAAACGCTYTCGCATC	16	53.1	48.9–50.8	This study
Gre1378R	CGTATTCATCGSAGACTG	18	50	49.6–50.4	This study

**Figure 5:**
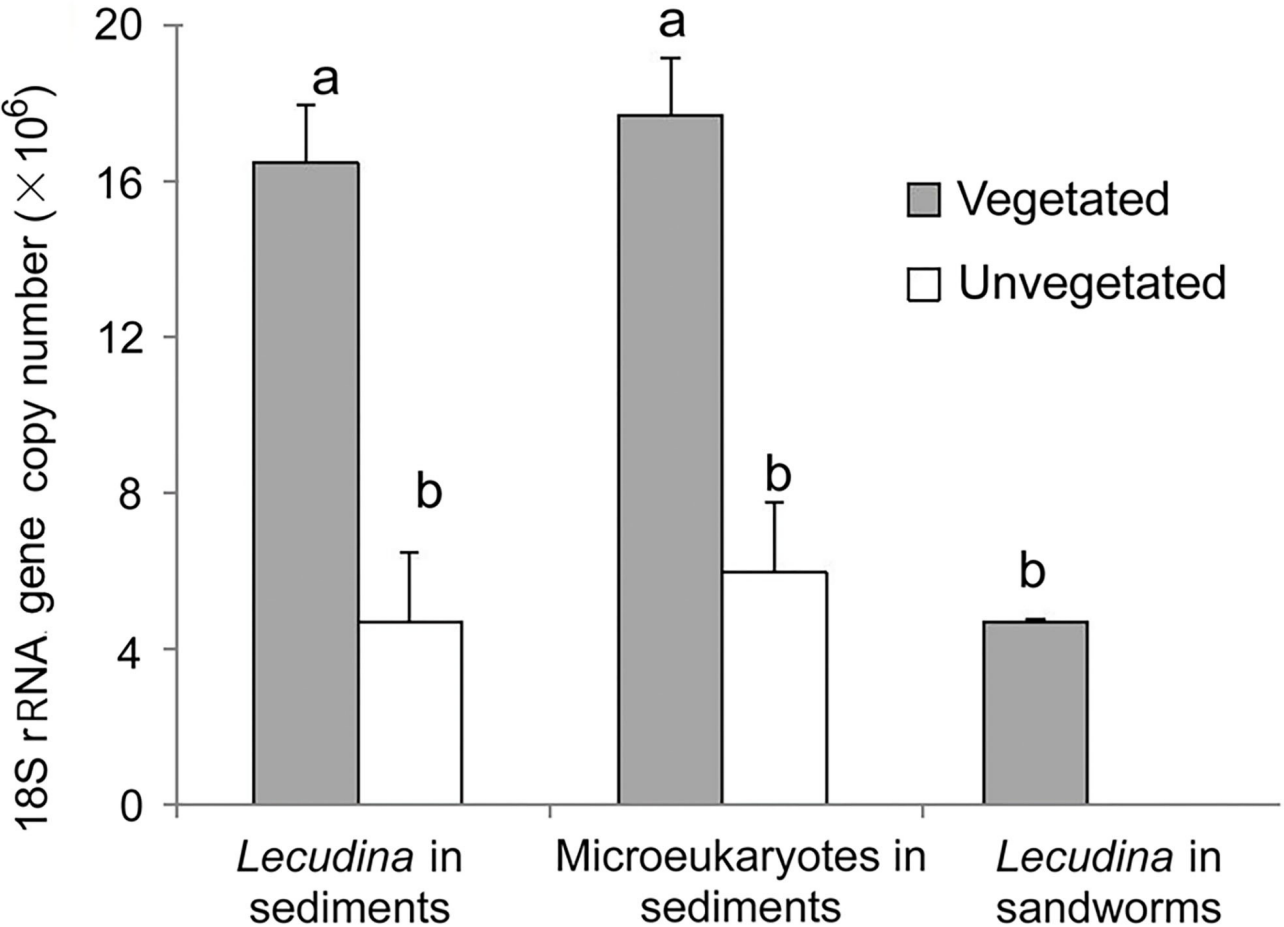
Comparisons of 18S rRNA gene copy numbers of *Lecudina polymorpha* and all microeukaryotes between the vegetated and unvegetated sediments, and in the whole-body tissue mixture of a sandworm species. Different labeling letters (a and b) indicate that the mean values of vegetated and unvegetated samples were significantly different by *t-*test.

The qPCR results demonstrated high 18S rRNA gene abundances of *Lecudina polymorpha* in both types of sediment samples (Figure 5). There were (1.65 ± 0.15) × 10^7^ copies g^−1^ in the vegetated samples, which was about three times higher than in the unvegetated ones [(4.7 ± 1.8) × 10^6^ copies g^−1^; *P* = 0.001, *n* = 5]. Application of the qPCR assay for DNA extracted from sandworm tissues succeeded, resulting in (4.7 ± 0.1) × 10^6^ copies g^−1^ (Figure 5), indicating the sandworms be the host of this gregarine parasite. According to the read proportions of the apicomplexan and gene abundance obtained using qPCR, the 18S rRNA gene abundances of all microeukaryotes were calculated, which showed higher abundance in the seagrass-colonized sediments [(17.7 ± 1.5) vs. (6.0 ± 2.0) × 10^6^ copies g^−1^] as well (Figure 5).

**Figure 6:**
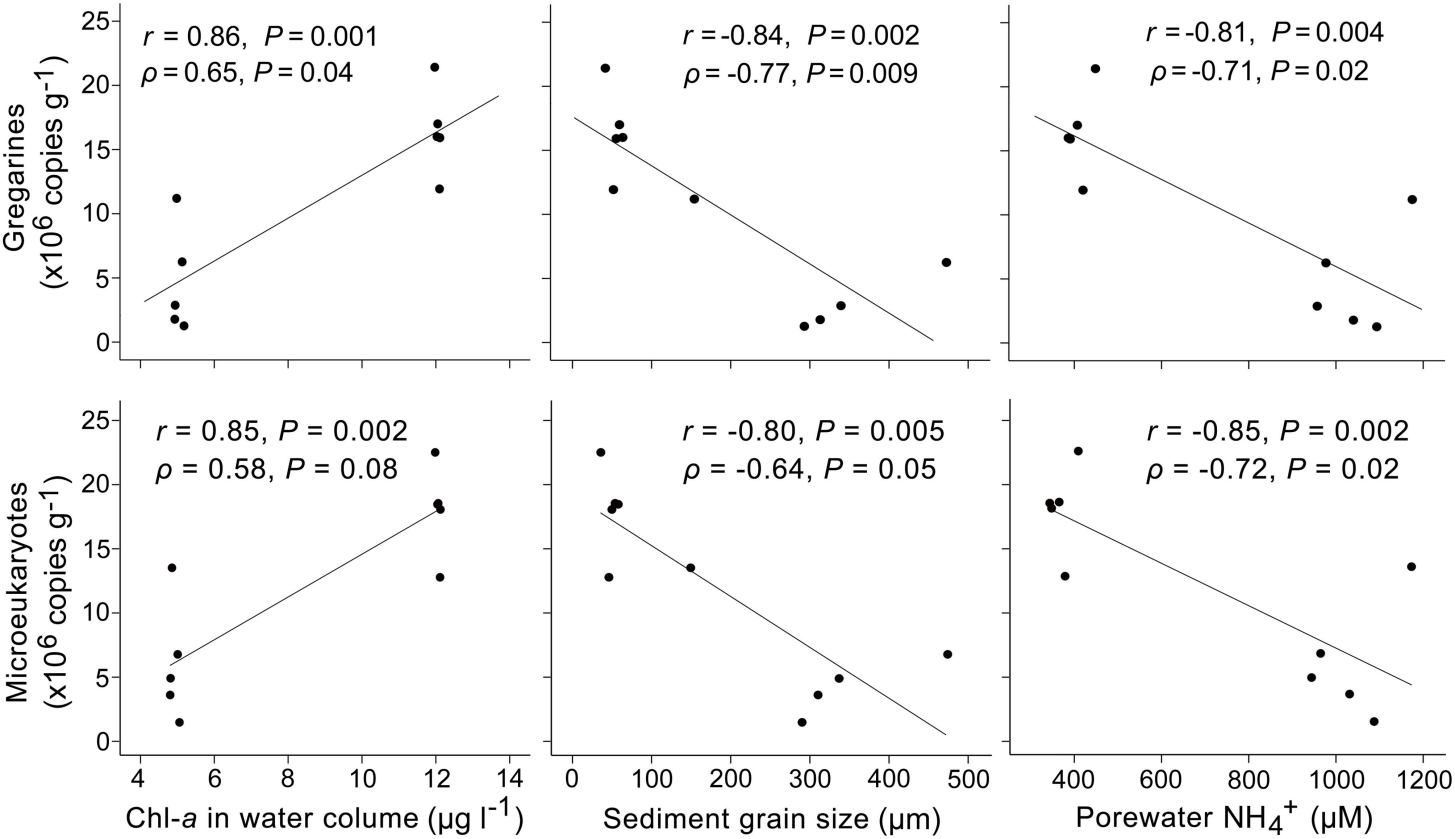
Correlations between the 18S rRNA gene abundances of gregarine apicomplexans, all microeukaryotes, and the measured environmental factors across vegetated and unvegetated sediments. The gene copy number of gregarines was significantly and positively correlated with the concentration of chlorophyll *a* in the overlying water and negatively with the sediment grain size and concentration of ammonium in sediments. Note that the gene copy number of microeukaryotes was estimated based on the gregarine abundance and their read proportion in the community.

Correlation analysis showed that *L. polymorpha* gene abundance was significantly and positively correlated with the concentration of chlorophyll *a* in the overlying water (*r* = 0.86, ρ = 0.65, *p* < 0.05) and significantly and negatively with the sediment grain size (*r* = −0.84, ρ = −0.77; *p* < 0.01) and the concentration of ammonium in sediments (*r* = −0.81, ρ = −0.71; *p* < 0.05) (Figure 6).

### Shifts in Functional Structure of Microeukaryotic Communities

**Figure 7:**
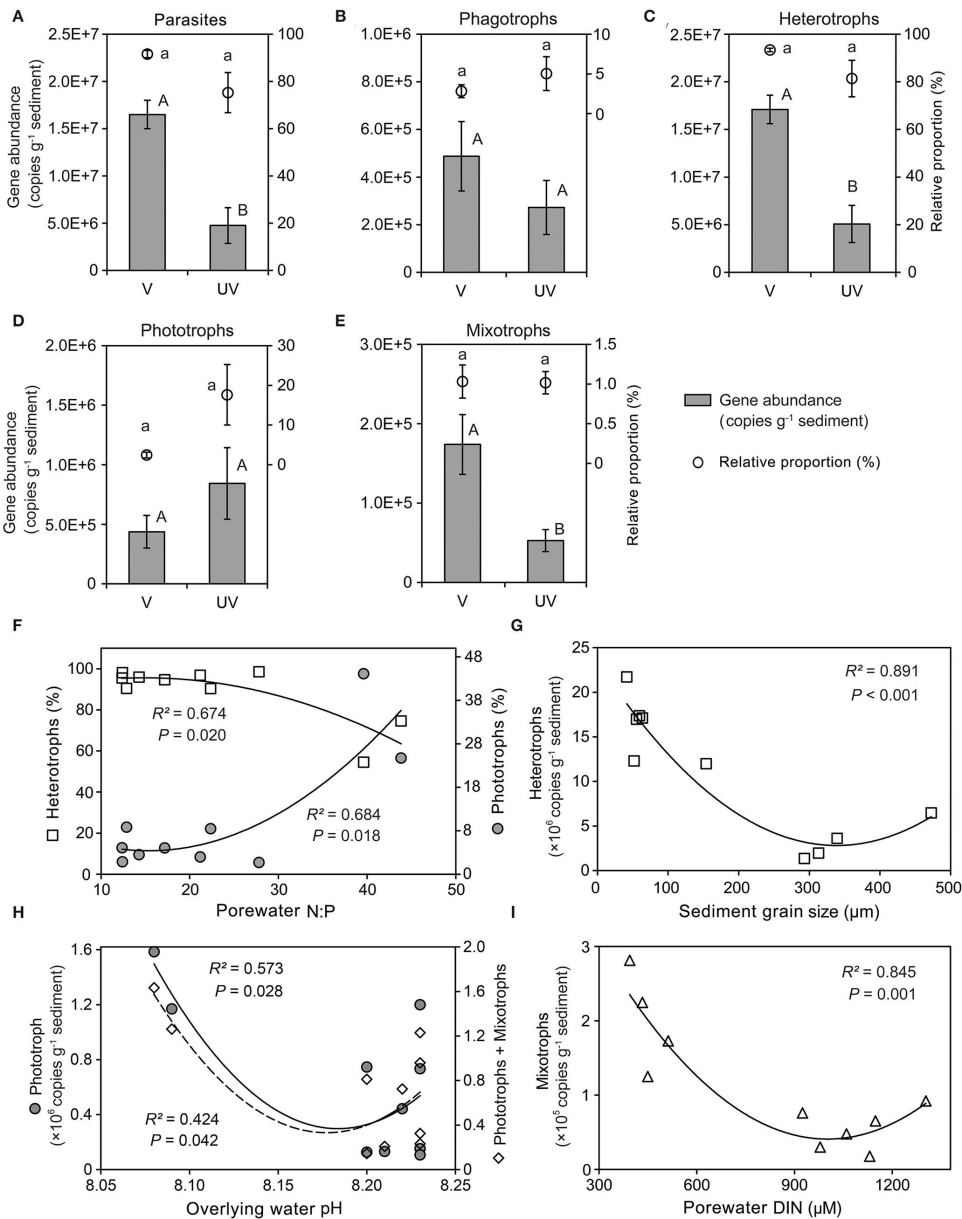
Functional compositions of benthic eukaryotes in sediments of a seagrass system. **(A–E)** Comparison of the relative proportions and gene abundances of parasites **(A)**, phagotrophs **(B)**, heterotrophs **(C)**, phototrophs **(D)**, and mixotrophs **(E)** between vegetated and unvegetated sediments, showing that the read proportions were not significantly different for all the examined functional groups, whereas the total gene copy numbers of parasites, heterotrophs, and mixotrophs were significantly higher in the vegetated sediments; **(F)** the relative proportions of both heterotrophs and phototrophs were related to molar ratio of porewater inorganic nitrogen to phosphate; **(G–I)** the gene abundances of heterotrophs, phototrophs (also pigmented protists), and mixotrophs covaried well with sediment grain size **(G)**, overlaying water pH **(H)**, and concentration of porewater dissolved inorganic nitrogen (DIN) **(I)**, respectively.

Both relative proportions and gene abundances of functional groups were statistically tested between habitats and along environmental gradients (Figure 7; Supplementary Figure S6). Between vegetated and unvegetated sediments, the differences in read proportions were detected for neither parasites, phagotrophs, heterotrophs, phototrophs, nor mixotrophs (all *P* < 0.05; Figures 7A–E). However, the gene copy numbers of functional groups (i.e., parasites, heterotrophs, and mixotrophs) were significantly higher in seagrass-colonized sediments than in bare sediments (*P* < 0.05; Figures 7A,C,E).

Among all the measured environmental factors, the molar ratio of dissolved inorganic nitrogen (DIN) to phosphate (N:P) was the most significant factor related to the read proportions of phototrophs (*R*^2^ = 0.684, *P* < 0.05) and heterotrophs (*R*^2^ = 0.674, *P* < 0.05) in the community (Figure 7F). Nevertheless, absolute abundance of heterotrophs formed a quadratic relationship with grain size (*R*^2^ = 0.891, *P* < 0.05), with the lowest abundance at sediment median grain size of 350 μm (Figure 7G). Similarly, both phototrophs (*R*^2^ = 0.573, *P* < 0.05) and pigmented eukaryotes (= phototrophs + mixotrophs; *R*^2^ = 0.424, *P* < 0.05) had the lowest gene copy numbers at an intermediate pH (8.17; Figure 7H). The mixotrophs became the least abundant at an intermediate level of DIN (ca. 1,000 μM; *R*^2^ = 0.845, *P* = 0.001; Figure 7I).

## Discussion

### Lower Diversity of Microbial Eukaryotes in Seagrass-Colonized Than in Bare Sediments

To our knowledge, this is the first study to compare eukaryotic microbial communities between seagrass-vegetated and unvegetated sediments using high-throughput sequencing technology. Our results showed that the unvegetated sediment harbored richer microbial eukaryotes than the vegetated one, which is different from the bacterial and archaeal communities, whose alpha diversity was not much different between these two habitats (Sun et al., 2015; Zheng et al., 2019). The different effects of seagrass colonization on alpha diversity of microbes suggest that microbial eukaryotes represent a significant module in benthic microbial food webs in response to environmental turbulence.

We found that the concentrations of several heavy metals (especially Cd) were significantly and negatively correlated with OTU richness of benthic microeukaryotes, suggesting higher concentrations of heavy metals could contribute to lower diversity of microeukaryotes in the vegetated sediments. This could be due to high sensitivity of eukaryotes to metal stress (Corcoll et al., 2019). A previous study on heavy metal pollution in the Swan Lake lagoon showed that the heavy metals (especially Cd) probably come from the use of phosphate fertilizer and corrosion-resistant painting of the fishing boats, as well as from the industrial activities of the adjacent city of Rongcheng Bay (Huang et al., 2013). Apart from this, dissolved oxygen (DO), a chemical factor not measured in this survey, could also be a factor leading to a lower diversity of microeukaryotes in the seagrass-colonized sediment. Due to DO penetration into sediments through seagrass roots at day and exclusive consumption of oxygen at night, there could be larger variability of redox status around the seagrass roots (than in the bare sediments), which may select for fewer protistan species that adapt to wider oxygen tensions (Fenchel and Finlay, 2008). In fact, the sediment near mangrove roots tends to have a lower richness of microbial eukaryotes as well (Zhu et al., 2018). In addition, the markedly higher read proportions of apicomplexan parasite OTUs in this study might have also contributed to the lower diversity of microbial eukaryotes in seagrass-colonized sediments.

### Effects of Seagrass on Taxonomic and Functional Composition of Benthic Microbial Eukaryotic Community

We found that seagrass significantly affected the taxonomic composition of microbial eukaryotes in the sediments. The concentration of porewater ${\text{NH}}_{4}^{+}$ was found to be an important abiotic factor explaining the microeukaryotic taxonomic differences between the vegetated and bare sediments. ${\text{NH}}_{4}^{+}$ is known as a dissolved inorganic nitrogen species regenerated from degradation of buried organic matter. Root uptake of ${\text{NH}}_{4}^{+}$ by the seagrass could lower nitrogen availability for epibenthic microalgae (Thornton et al., 1999) and further affect their species richness and composition, which was partly supported by our result that autotrophic chlorophytes were identified to be a major contributor to the community differences (Supplementary Table S1). Another mechanism might be enhancement of ammonia oxidation by nitrifiers in root-associated sediments due to oxygen release through root (Sun et al., 2015; Lin et al., 2021), which resulted in structure shift of nitrogen species (i.e., more nitrate and less ammonium), thus modulating the microalgal composition of different nitrogen assimilatory preference (Dortch, 1990).

From a functionally ecological point of view, we identified significant effects of seagrass colonization on benthic microbial eukaryotes as well. According to recent findings on positive relationships between 18S rRNA gene copy number and protistan biomass (biovolume) (Fu and Gong, 2017; Zou et al., 2021), our comparisons of absolute gene abundances indicate a higher biomass of heterotrophic microeukaryotes (mostly gregarine apicomplexan parasites) in seagrass-colonized (fine) vs. bare (coarse) sediments. This is consistent with previous studies showing higher abundance of benthic animal hosts of these parasites (i.e., polychaetes; see below) at the sites with a higher seagrass biomass and finer sediments (Omena and Creed, 2004; Abrogueña et al., 2021). Apart from the parasites, tintinnid ciliates were identified to have higher biomass at the vegetated sites (Supplementary Table S2), indicating that free-living protozoa contributed to the functional shift in microeukaryotic community. Nevertheless, understanding the direct cause of higher planktonic ciliates in seagrass-associated sediments needs more details, for example, whether these ciliates present at an inactive life cycle stage (cysts), relic DNA, and whether they inhabit surface sediments warrant further investigation (Zhu et al., 2018; Zou et al., 2021).

It is interesting to observe that the gene abundance of benthic microphytobenthos (= phototrophs + mixotrophs) forms a U-shaped relationship with pH of overlying waters, instead of nutrient concentrations in waters or sediments (Figure 7H). This result is in line with the study of microphytobenthos in seagrass systems in south Florida (Fourqurean et al., 2010). Our previous characterization of environmental variables across the studied sites showed that both chlorophyll *a* concentration (average 12.1 vs. 5.0 μg/L) and pH value (average 8.22 vs. 8.16) in overlying waters were higher in seagrass sites (Sun et al., 2015), suggesting that spatial heterogeneity of seawater pH was shaped by photosynthetic activity and biomass of seagrass and phytoplankton, which increase pH by absorbing dissolved CO_2_. The U-shaped relationship suggests that there were different processes controlling the growth of microphytobenthos along the pH gradient. At certain bared sites with a distance from the seagrass bed, there were no seagrass canopy and roots competing for sunlight and regenerated nitrogen, which could promote the growth of microphytobenthos, leading to the highest autotrophic biomass at the lowest pH (left side of the U-shaped curve). At the vegetated sites, microphytobenthos might suffer from competing with abundant seagrass, which corresponded to the upper range of pH. However, epiphytic microalgae detached from seagrass leaves may settle to sediment surface (Bologna and Heck, 1999) and thus supplied and maintained a high level of autotrophic biomass, which explains the right side of the U-shaped curve. The lowest level of benthic autotrophs could be from the bare sites near seagrass or vegetated sites of lower seagrass density.

There were no significant differences in read proportions of functional groups within benthic microeukaryotic communities between vegetated and unvegetated sites, highlighting a degree of functional redundancy of benthic microeukaryotic community across habitat boundaries. This could be partly due to export of particulate (e.g., seagrass leaves, and seagrass-associated and macroalgal detritus) and dissolved organic matter from vegetated to near and distant unvegetated locations (Heck et al., 2008). Furthermore, it has been demonstrated that the effect of seagrass on macrofauna could extend to bare sites at a distance of about 5 m (Han et al., 2017). Therefore, both energy and nutrient transportation and biotic interactions with higher trophic levels could weaken the cross-habitat distinctness in microeukaryotic functionality. Nevertheless, our result indicates that porewater N:P ratio could be a good indicator for the contributions of heterotrophs and autotrophs to benthic microeukaryotic community across vegetated and unvegetated sediments. A mechanistic understanding to this association may involve complicated physical, ecological, and stoichiometric interactions among seagrass, benthic animals, prokaryotes, and the microeukaryotes, which needs further investigation. Recently, a similar result on the importance of nutrients in driving functional composition has been reported for small planktonic eukaryotes (Wang et al., 2020).

### Seagrass Bed as a Marine Hotspot of Gregarine Apicomplexans

For the first time, our study demonstrates that the seagrass bed represents a hotspot of Apicomplexa, which represented on average 83% reads across vegetated and unvegetated sediments. Hosts of apicomplexan parasites are known as man, domestic and wild animals, and invertebrates (Levine, 2018). Metabarcoding projects of microeukaryotes or protists in marine ecosystems have generally revealed low proportions of these parasites. For example, Apicomplexa accounted for on average 2.5% and up to 10% reads in surface sediments across Bohai Sea and Yellow Sea (Gong et al., 2015), up to 20% in mangrove sediments (Zhu et al., 2018), and <5% in coastal and oceanic waters (see Mahé et al., 2017). However, in terrestrial ecosystems, moderate amounts of apicomplexans have also been detected in protistan surveys in world's soils (e.g., few to more than 50%, Bates et al., 2013), and comparable abundances (average 84%) in the Neotropical rainforest soils (Mahé et al., 2017). These suggest that, by and large, apicomplexans are widespread in soil and sedimentary habitats, especially highly productive systems. This abundance hotspot is apparently linked to a generally high biomass of their hosts dwelling in these belowground environments (see our discussion above).

Based on phylogenetic analysis and qPCR results, we confirm the apicomplexan phylotypes obtained in this study are closely related to *Lecudina polymorpha* morphotype 2, a gregarine morphospecies known to parasitize and an unidentified species of *Lumbrineris* (Leander et al., 2003). These results are consistent with existing knowledge, as Polychaeta (including *Lumbrineris latreilli*) dominate the macrobenthic community in the seagrass meadow of Swan Lake (Han et al., 2017), and all *Lecudina* species are known to inhabit intestine of polychaetes (Levine, 2018). In fact, *Lecudina polymorpha* Schrével, 1963 parasitizes the sandworm *Lumbrineris latreilli* (see Levine, 2018), and related *Lecudina* phylotypes have been detected in environmental DNA extracted from mangrove sediments as well (Zhu et al., 2018). These parasites can also be released into the environment with host feces in the form of mature sporocysts (oocyst). Much higher abundance of gregarine 18S rRNA gene was detected in sediment than in sandworm body, suggesting high abundance of Gregarine sporocysts accumulated in the sediment of seagrass bed, and new hosts might get infected when an oocyst is ingested and sporozoites escape into their digestive tract (Solter et al., 2012).

Due to differences in vertebrate and invertebrate diversity and composition of host communities, it is reasonable to detect different groups of apicomplexans across global soils, that is, Eucoccidiorida (Bates et al., 2013) and diverse species of gregarines (Mahé et al., 2017). So far, gregarine apicomplexans have been only reported from 0.32% of invertebrate species (Levine, 2018). There is a well-known high diversity of benthic invertebrates in coastal and deep oceans (Dong et al., 2021), highlighting that a great diversity of gregarines/apicomplexans awaits to be unveiled and linked to their hosts, as we have shown in this study.

In sum, this study demonstrates that there are significant ecological effects of a seagrass on the diversity and community organization of belowground microbial eukaryotes. Seagrass colonization gives rise to a less diverse and more heterotrophic microeukaryotic community. The dominance of gregarine apicomplexan parasites of polychaetes or other macro-organisms in the benthic microeukaryotic community is probably a characteristic for high productivity of those local habitats, reflecting that the belowground microbial diversity is tightly linked with plants and animals through complex food webs in the coastal environment.

## Data Availability Statement

The data presented in the study are deposited in the NCBI repository, accession number PRJNA797205, and OM925574 to OM925753.

## Author Contributions

YP: writing—original draft and editing. GL: data curation and figure plotting. LS: data curation and sample collection. PZ and YW: sample collection. ZS: revision and supervision. ZC: revision. QH: supervision. JG: project administration and writing—review and editing. All authors contributed to the article and approved the submitted version.

## Funding

This work was supported by the National Natural Science Foundation of China (Nos. 41976128 and 41906113), the Natural Science Foundation of Jiangsu Province (No. BK20190494), the Innovation Group Project of Southern Marine Science and Engineering Guangdong Laboratory (Zhuhai) (No. 311021004), and the CAS Frontiers in Scientific Research Programs, Projects for Young Top-notch Talents (QYZDB-SSW-DQC013).

## Conflict of Interest

The authors declare that the research was conducted in the absence of any commercial or financial relationships that could be construed as a potential conflict of interest.

## Publisher's Note

All claims expressed in this article are solely those of the authors and do not necessarily represent those of their affiliated organizations, or those of the publisher, the editors and the reviewers. Any product that may be evaluated in this article, or claim that may be made by its manufacturer, is not guaranteed or endorsed by the publisher.
