# Supplementary material for: Seagrass Colonization Alters Diversity, Abundance, Taxonomic, and Functional Community Structure of Benthic Microbial Eukaryotes
Source: Front Microbiol. 2022 Jun 13;13:901741. doi: 10.3389/fmicb.2022.901741 (PMC9234489; doi:10.3389/fmicb.2022.901741)
Supplement: Supplementary file 1 [file Data_Sheet_1.docx]

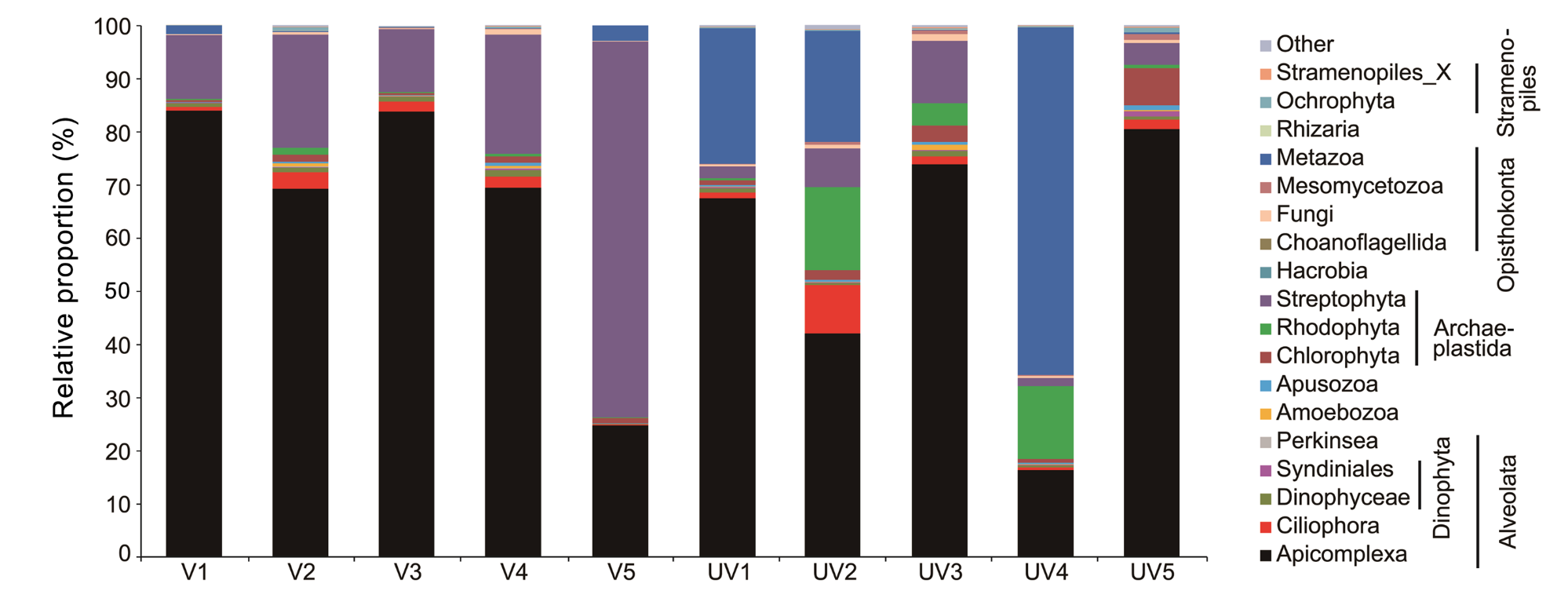


Fig. S1. Relative proportions of reads for benthic eukaryotes (including macroorganisms such as Metazoa and Streptophyta) across vegetated (V1-V5) and unvegetated (UV1-UV5) sites of the seagrass ecosystem.


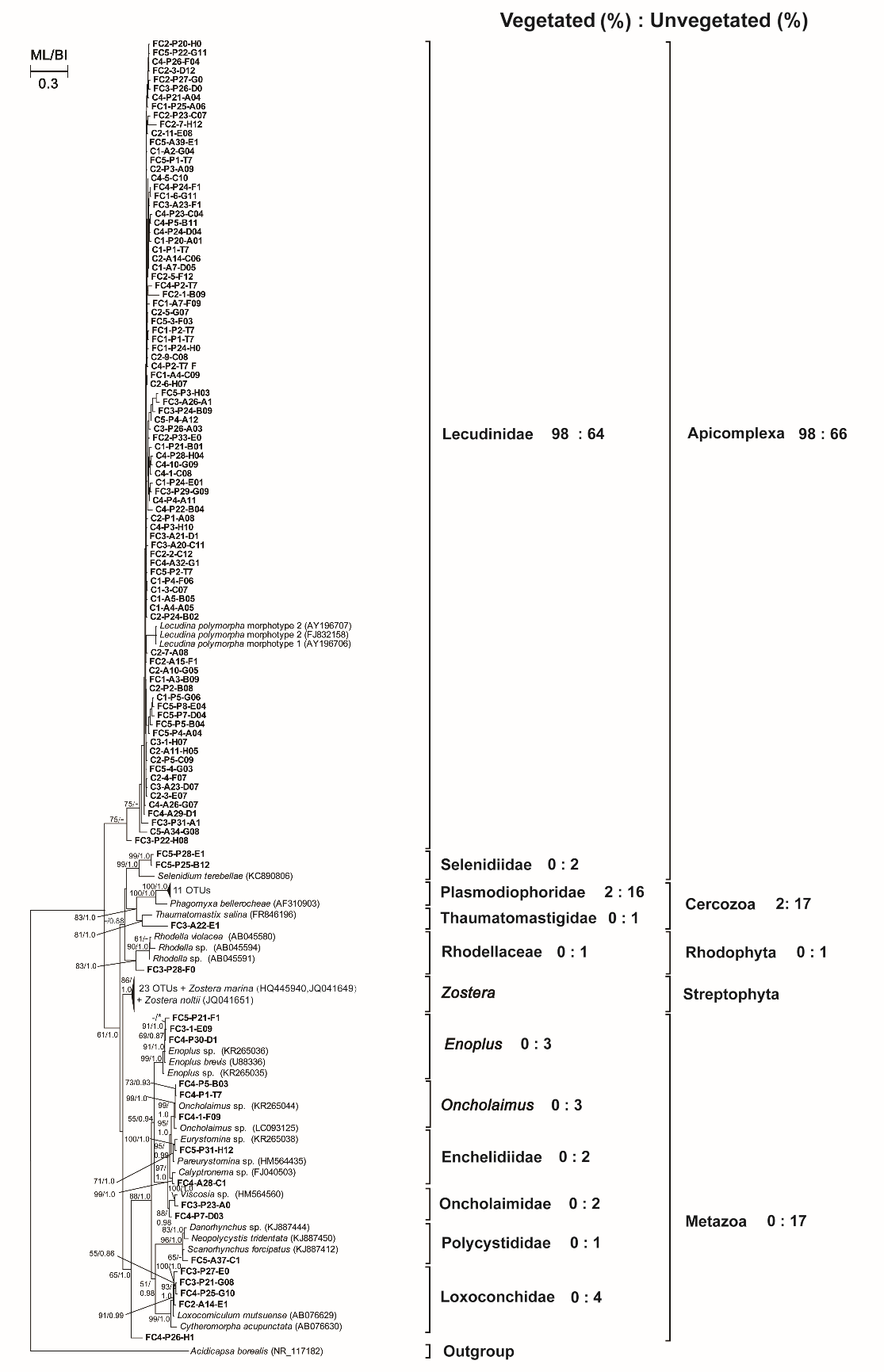


Fig. S2. Correlations between OTU richness of microeukaryotes and concentrations of metals (As, Cu, Cr, Pb, Ni, Co, Fe), chlorophyll *a* (Chl-*a*) and ammonium (NH_4_^+^) in sediments.


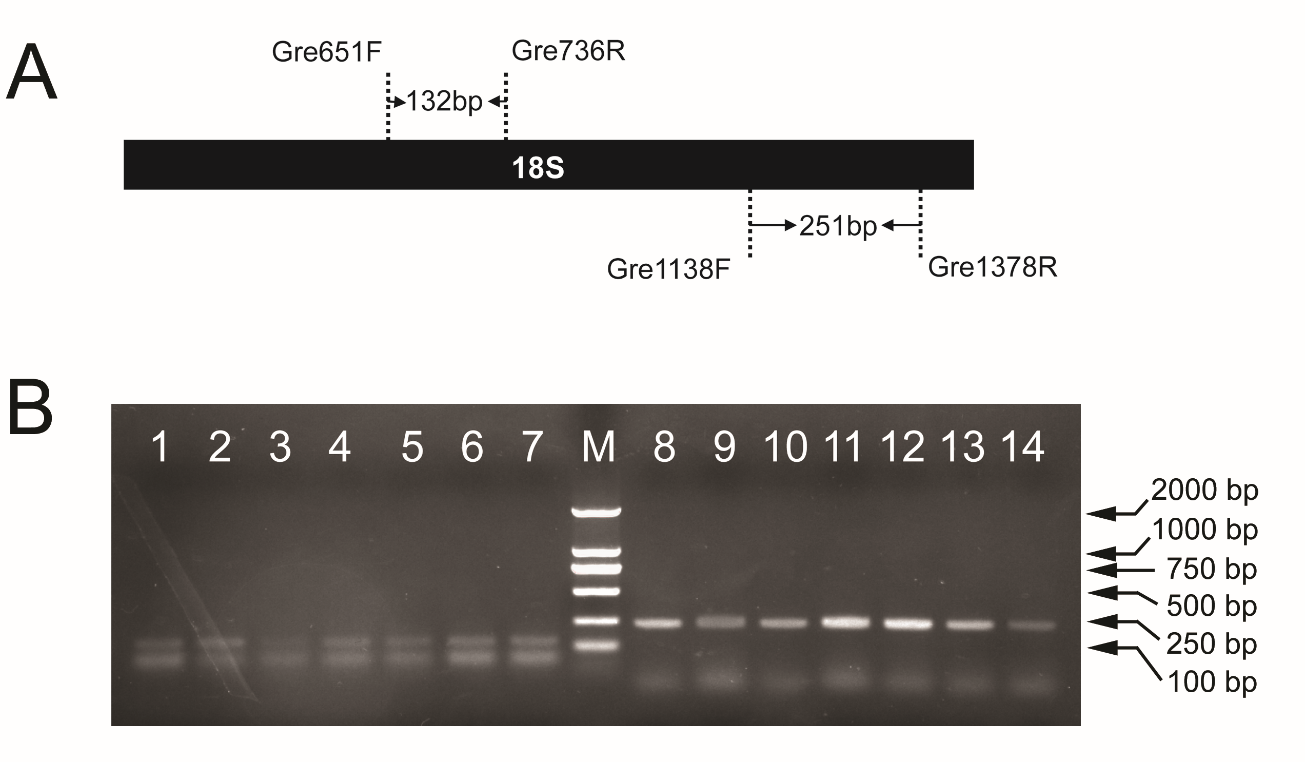


Fig. S3. A phylogenetic tree based on 18S rRNA gene using maximum likelihood (ML) and Bayesian inference (BI) algorithms. The sequence proportions of major taxonomic groups in vegetated and unvegetated sediments were shown. Only bootstrap values higher than 50% or posterior probability higher than 0.8 were given at the nodes. Dashes (-) indicate lower values than the set standards. Asterisks indicate alternate topologies.


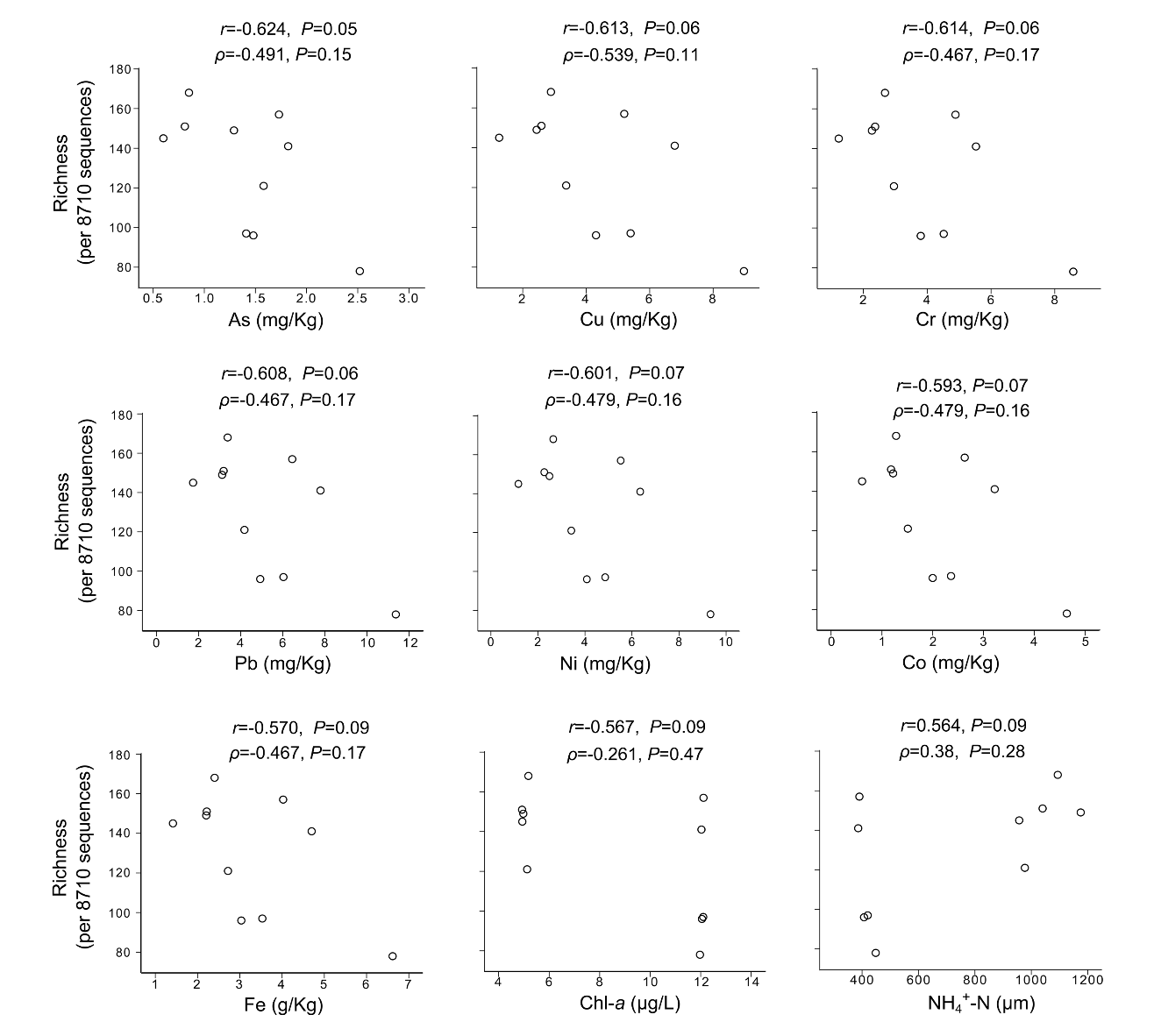


Fig. S4. (A) Schematic diagram showing the regions of 18S rRNA gene targeted by the newly designed specific primers for the gregarines detected in this study; (B) A electrophoresis image showing PCR products using the DNA extracted from sandworms with primers Gre651F-Gre736R (lane 1-7) and Gre1138F-Gre1378R (lane 8-14). Lane M indicates DNA ladders.


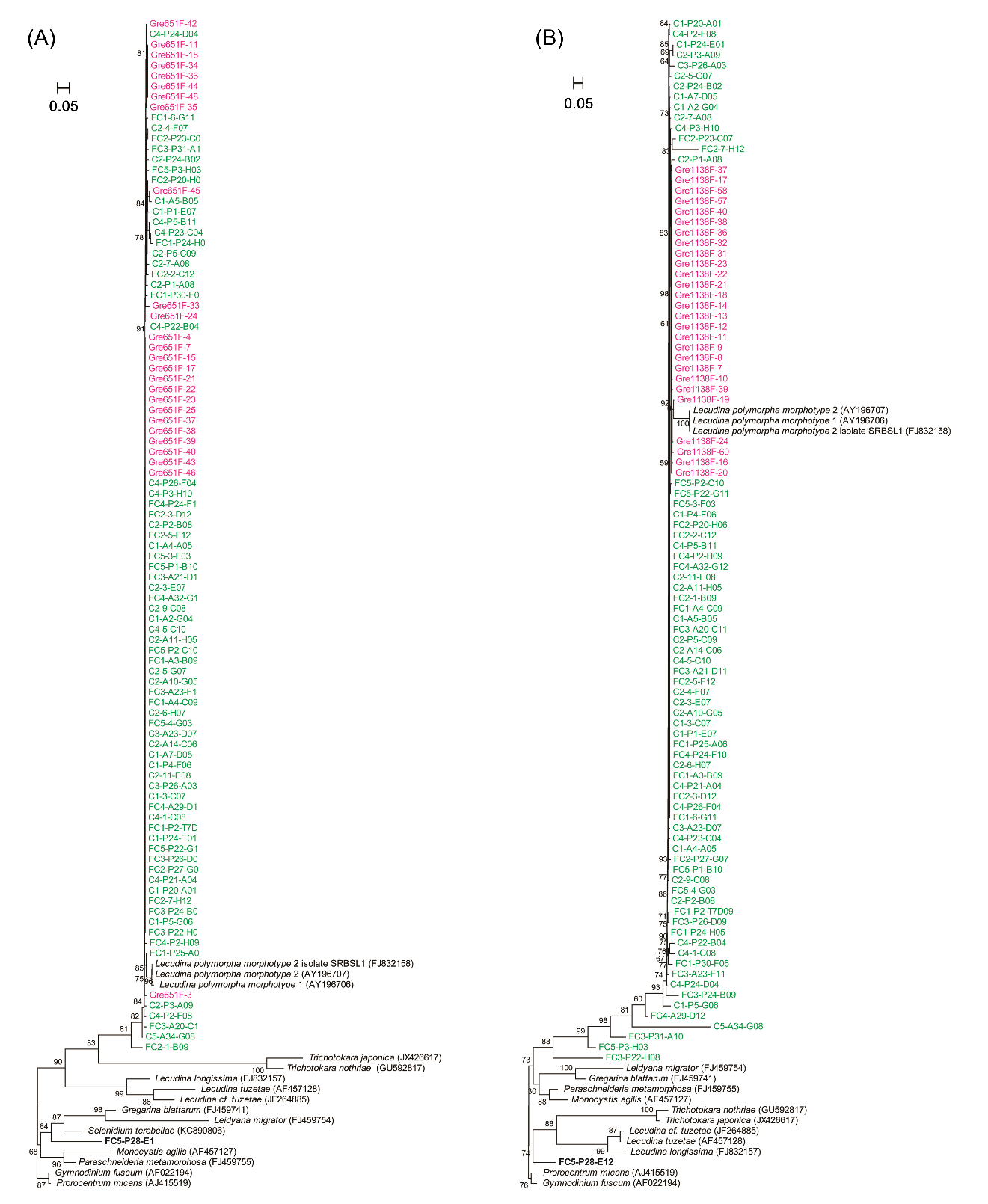


Fig. S5. A maximum-likelihood tree confirming the specificity of the newly designed qPCR primers Gre651F-Gre736R (A) and Gre1138F-Gre1378R (B). Note that the sequences derived from qPCR assays (in pink) are well nested within the longer sequences obtained from the clone libraries and Sanger’s sequencing (in green). Only bootstrap values higher than 50 were given at the nodes.


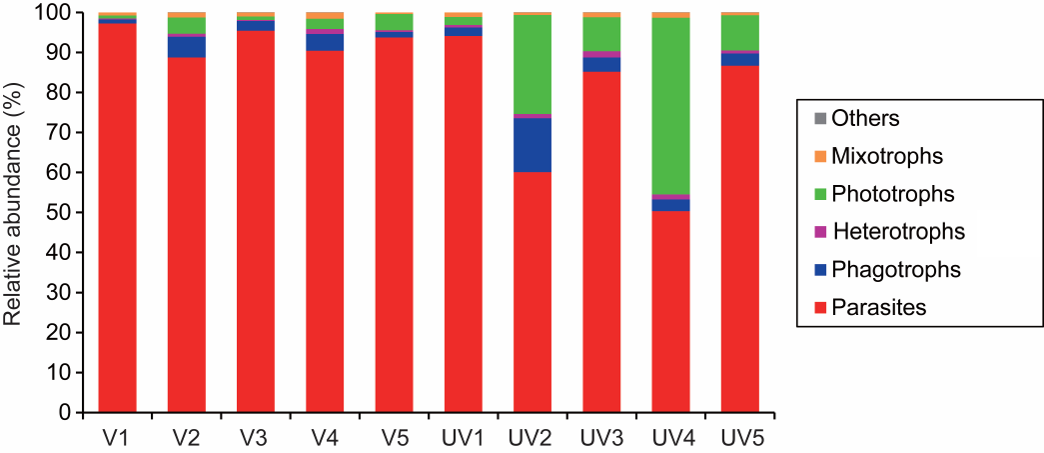


Fig. S6. Compositional profiles of functional groups of microbial eukaryotes, including parasites, phagotrophs, heterotrophs, phototrophs, mixotrophs and others across vegetated (V1-V5) and unvegetated (UV1-UV5) sites of the seagrass ecosystem.

**Table S1.** Contributions of taxonomy groups to the difference in microeukaryotic community structure between seagrass vegetated and unvegetated sediments.

| Group | Vegetated versus Unvegetated | | |
| --- | --- | --- | --- |
|  | Average abundance % (min - max) | Richness | Contribution % |
| Fungi | 0.8 (0.2-1.3) | 87 | 16.48 |
| Chlorophyta | 2.4 (0.4-7.3) | 66 | 14.67 |
| Dinophyta | 1.2 (0.4-1.9) | 55 | 12.68 |
| Ciliophora | 3 (0.8-12.6) | 41 | 12.25 |
| Apicomplexa | 83.2 (49.4-97) | 30 | 9.58 |
| Stramenopiles_X | 0.1 (0.0-0.2) | 41 | 5.77 |
| Breviatea | 0.3 (0.0-0.7) | 23 | 5.50 |
| Ochrophyta | 0.4 (0.1-0.9) | 20 | 4.94 |
| Rhodophyta | 7.2 (0.2-41.5) | 15 | 2.59 |
| Mesomycetozoa | 0.4 (0.0-1.2) | 9 | 2.04 |
| Apusomonadidae | 0.5 (0.1-0.9) | 8 | 1.9 |
| Cercozoa | 0 | 14 | 1.81 |
| Perkinsea | 0 | 2 | 0.57 |
| Haptophyta | 0 | 3 | 0.53 |
| Choanoflagellida | 0 | 1 | 0.17 |
| Centroheliozoa | 0 | 1 | 0.11 |
| Others | 0.4 | 56 | 8.59 |

**Table S2.** Shifts in relative abundance of major microeukaryotic taxa in the vegetated and unvegetated sediments.

| Taxonomy | % Relative abundance（mean ± SE）in： | | | 18S rRNA gene copy number (x10^6^) g^-1^ sediment（mean ± SE）in： | | |
| --- | --- | --- | --- | --- | --- | --- |
|  | Vegetated | Unvegetated | *P* | Vegetated | Unvegetated | *P* |
| **Alveolata** | 95.92±1.08 | 78.92±7.70 | 0.09 | **17.02 ± 1.19** | **4.97 ± 1.89** | **<0.01** |
| Apicomplexa, Apicomplexa_X | 92.66±1.65 | 73.78±8.40 | 0.09 | **16.45 ± 1.51** | **4.67 ± 1.85** | **<0.01** |
| Gregarines | 91.96±1.81 | 52.12±14.24 | **0.05** | **16.32 ± 1.51** | **4.03 ± 1.97** | **<0.01** |
| Apicomplexa_XX | 0.66±0.27 | 21.50±10.86 | 0.13 | **0.12 ± 0.05** | **0.64 ± 0.18** | **0.04** |
| Perkinsea, Perkinsida | 0.04±0.02 | 0.00±0.00 | 0.18 | 0.01 ± 0.00 | 0.00 ± 0.00 | 0.21 |
| Ciliophora | 2.08±0.58 | 3.84±2.19 | 0.47 | 0.36 ± 0.11 | 0.21 ± 0.11 | 0.36 |
| Spirotrichea | 2.08±0.58 | 3.84±2.19 | 0.47 | 0.36 ± 0.11 | 0.21 ± 0.11 | 0.36 |
| Oligotrichia | 1.14±0.28 | 1.86±0.69 | 0.38 | 0.20 ± 0.05 | 0.11 ± 0.04 | 0.18 |
| Choreotrichia | 0.90±0.31 | 1.96±1.52 | 0.53 | 0.16 ± 0.05 | 0.10 ± 0.07 | 0.57 |
| Strombidinopsidae | 0.12±0.06 | 1.72±1.55 | 0.36 | 0.02 ± 0.01 | 0.08 ± 0.08 | 0.45 |
| Tintinnidae | 0.18±0.06 | 0.02±0.02 | **0.05** | **0.03 ± 0.01** | **0.00 ± 0.00** | **0.03** |
| Dinophyta | 1.14±0.25 | 1.30±0.16 | 0.61 | 0.20 ± 0.05 | 0.08 ± 0.04 | 0.09 |
| Dinophyceae | 0.98±0.22 | 0.92±0.13 | 0.82 | **0.17 ± 0.04** | **0.05 ± 0.01** | **0.02** |
| Dinophyceae_X | 0.70±0.15 | 0.66±0.11 | 0.83 | **0.12 ± 0.03** | **0.03 ± 0.01** | **0.02** |
| Suessiales | 0.26±0.06 | 0.28±0.04 | 0.79 | **0.05 ± 0.01** | **0.02 ± 0.01** | **0.03** |
| Syndiniales | 0.16±0.07 | 0.38±0.16 | 0.25 | 0.03 ± 0.01 | 0.04 ± 0.03 | 0.74 |
| **Amoebozoa**, Breviatea | 0.28±0.14 | 0.36±0.19 | 0.74 | 0.05 ± 0.02 | 0.01 ± 0.00 | 0.17 |
| **Apusozoa**, Apusomonadidae | 0.34±0.13 | 0.64±0.09 | 0.10 | 0.06 ± 0.02 | 0.04 ± 0.02 | 0.57 |
| **Archaeplastida** | 2.14±0.68 | 17.6±7.85 | 0.12 | 0.39 ± 0.12 | 0.81 ± 0.29 | 0.24 |
| Chlorophyta | 1.52±0.58 | 3.36±1.05 | 0.17 | 0.28 ± 0.11 | 0.26 ± 0.18 | 0.94 |
| Ulvophyceae | 0.68±0.49 | 1.36±0.96 | 0.55 | 0.12 ± 0.09 | 0.15 ± 0.14 | 0.87 |
| Chlorophyceae | 0.36±0.09 | 1.02±0.25 | 0.06 | 0.06 ± 0.02 | 0.05 ± 0.01 | 0.65 |
| Trebouxiophyceae | 0.26±0.05 | 0.54±0.07 | **0.02** | 0.04 ± 0.01 | 0.04 ± 0.02 | 0.74 |
| Rhodophyta | 0.60±0.28 | 13.84±7.95 | 0.17 | 0.11 ± 0.05 | 0.55 ± 0.31 | 0.19 |
| **Opisthokonta** | 0.66±0.20 | 1.64±0.26 | **0.02** | 0.11 ± 0.04 | 0.09 ± 0.04 | 0.70 |
| Fungi | 0.58±0.20 | 1.00±0.16 | 0.12 | 0.10 ± 0.03 | 0.05 ± 0.01 | 0.18 |
| Ascomycota | 0.28±0.11 | 0.36±0.09 | 0.58 | 0.05 ± 0.02 | 0.02 ± 0.01 | 0.17 |
| Pezizomycotina | 0.26±0.11 | 0.36±0.09 | 0.50 | 0.05 ± 0.02 | 0.02 ± 0.01 | 0.25 |
| Chytridiomycota | 0.10±0.04 | 0.42±0.14 | 0.08 | 0.02 ± 0.01 | 0.02 ± 0.01 | 0.95 |
| Basidiomycota | 0.04±0.02 | 0.08±0.04 | 0.40 | 0.01 ± 0.00 | 0.00 ± 0.00 | 0.15 |
| Mesomycetozoa | 0.08±0.04 | 0.64±0.18 | **0.04** | 0.01 ± 0.01 | 0.05 ± 0.03 | 0.29 |
| **Rhizaria**, Cercozoa | 0.04±0.02 | 0.06±0.04 | 0.68 | 0.01 ± 0.00 | 0.00 ± 0.00 | 0.25 |
| **Stramenopiles** | 0.14±0.07 | 0.48±0.17 | 0.54 | 0.07 ± 0.03 | 0.04 ± 0.03 | 0.49 |
| Ochrophyta | 0.32±0.11 | 0.38±0.13 | 0.73 | 0.05 ± 0.02 | 0.03 ± 0.02 | 0.55 |
| Bacillariophyta | 0.20±0.06 | 0.14±0.02 | 0.42 | 0.04 ± 0.01 | 0.01 ± 0.00 | 0.08 |
| Phaeophyceae | 0.08±0.06 | 0.12±0.12 | 0.77 | 0.01 ± 0.01 | 0.02 ± 0.02 | 0.79 |
| Stramenopiles_X | 0.10±0.03 | 0.16±0.02 | 0.17 | 0.02 ± 0.00 | 0.01 ± 0.00 | 0.27 |
| Labyrinthulea | 0.02±0.02 | 0.06±0.02 | 0.24 | 0.01 ± 0.00 | 0.00 ± 0.00 | 0.30 |
| MAST | 0.02±0.02 | 0.08±0.02 | 0.07 | 0.01 ± 0.00 | 0.00 ± 0.00 | 0.35 |
| **Others** | 0.04±0.02 | 0.14±0.04 | 0.07 | 0.01 ± 0.00 | 0.02 ± 0.00 | 0.78 |
